# RAP44 phage integrase-guided 50K genomic island integration in *Riemerella anatipestifer*

**DOI:** 10.3389/fvets.2022.961354

**Published:** 2022-11-29

**Authors:** Ying Wang, Jianfeng Deng, Jianle Ren, Libin Liang, Junping Li, Sheng Niu, Xingchen Wu, Yujun Zhao, Shimin Gao, Fang Yan, Yuqing Liu, Haili Ma, Wen-xia Tian, Yi Yan

**Affiliations:** ^1^College of Veterinary Medicine, Shanxi Agricultural University, Jinzhong, China; ^2^Institute of Animal Science and Veterinary Medicine, Shandong Academy of Agricultural Sciences, Jinan, China

**Keywords:** *Riemerella anatipestifer*, RAP44 phage, integrase, genomic island, integration

## Abstract

Bacteriophages are viruses that infect bacteria. Bacteria and bacteriophages have been fighting for survival. Over time, the evolution of both populations has been affected. Pathogenic Flavobacteriaceae species including *Riemerella anatipestifer* mainly infects ducklings, geese, and turkeys. However, it does not infect humans, rats, or other mammals, and is a suitable and safe research object in the laboratory. Our previous study showed that there is a 10K genomic island in *R. anatipestifer*In this study, we found another integrated 50K genomic islands and focused on the relationship between *R. anatipestifer* genomic islands and the RAP44 phage genome. The phage RAP44 genome was integrated into *R. anatipestifer* chromosome, and an evolutionary relationship was evident between them in our comparative analysis. Furthermore, the integrated defective RAP44 phage sequence had the function of integration, excision, and cyclization automatically. Integrases are important integration elements. The integrative function of integrase was verified in *R. anatipestifer*. The integrase with the attP site can be integrated stably at the attB locus of the *R. anatipestifer* genome. A recombinant strain can stably inherit and express the exogenous gene. By studying the integration between host bacterium and phage, we have provided evidence for the evolution of the genomes in *R. anatipestifer*.

## Introduction

Bacteriophages are viruses that infect bacterial cells. However, bacteria and bacteriophages are fighting for survival; over time, the evolution of both populations has been affected (1–3). It is possible to divide bacteriophages into temperate phages (prophages) and virulent phages (lytic bacteriophages) based on the replication process and survival status of the host bacteria.

Prophages are the main contributors to bacterial genetic diversity (4), and most vertically inherited prophage sequences are highly conserved. A large part of bacterial genome sequences is derived from prophages, and the domestication of prophages can promote the adaptability of bacteria. Approximately, 300 complete and defective prevention fragments were found in the intestinal bacterial genome. The sizes of these fragments showed a bimodal distribution; one peak represented a fully functioning prophage, ranging in size from 30 to 70 kb, and the other peak represented a defective prophage, ranging in size from 5 to 30 kb (4). Prophages are nucleic acids containing mild phages integrated into the host genome. Fully functioning prophages have excision, virion formation, lysis, or infective abilities. Prophages lacking these functions are referred to as defective prophages. Defective prophages can be beneficial to bacteria by protecting them from further bacteriophage infection and improving their antibiotic tolerance. Bacteria can also benefit from fully functional prophages. In *Listeria monocytogenes*, prophage excision promotes the expression of functional genes and enhances phagosomal escape and bacterial virulence (5). The prophage inserted into the bacterial chromosome has two outcomes: one is through a replicating lytic life cycle, and the other is that the prophage genome may become part of the bacterial chromosome through progressive mutations of critical genes or sites.

Prophages significantly influence the phenotype and pathogenicity of bacteria. For example, the integration of prophages results in the interruption or ectopic coding of host genes, leading to changes in the host phenotype. Similarly, the insertion of prophages introduces new encoded genes to enhance bacterial adaptability to the environment and pathogenicity to the host. The lysogenic conversion of prophages was also regulated by new bacterial phenotype. Therefore, prophages may be the main factors in horizontal gene transfer (HGT), evolution, and genetic diversity in bacteria (6). Penadés et al. recently reported a new family of pathogenic islands, known as phage-induced chromosomal islands. These islands are ubiquitous in bacteria and play an important role in the HGT, adaptability, and virulence of bacteria (7, 8). *Staphylococcus aureus* pathogenicity islands (SaPIs), also known as bacteriophage satellites, represent this new pathogenic island family. SaPIs are closely related to prophages because they exist throughout the life cycle of phages. SaPIs encode integrases (int) (9) and exonucleases (xis) (10) and can be removed from bacterial genomes to form large plasmid-like closed-loop DNA (11). Closed-loop DNA can then be packaged into infectious bacteriophage particles. These infectious particles are released by bacterial lysis and can adsorb and import SaPI DNA into the bacteria. The HGT can be accomplished by integrase-guided insertion into host chromosomes or replication of plasmids.

Almost half of the sequenced bacterial genomes contain complete prophages (12). There is a prophage named PP3 in the clinical isolates of *Pseudomonas aeruginosa* PAO1. PP3 can spontaneously be removed from the PAO1 chromosome at a frequency of approximately 25%. However, no phage plaque was formed in *P. aeruginosa* by induction, and no phage particles were detected in the supernatant; therefore, it was defined as a defective prophage. PP3 can be transferred to other *P. aeruginosa* strains. The first step after the transfer is integrating DNA into the bacterial chromosome. PP3 can be integrated into the same position as PAO1 and maintain circulatory capacity for integration and excision (13).

The pathogenic Flavobacteriaceae species including *Riemerella anatipestifer* mainly infects ducklings, geese, and turkeys. It does not infect humans, rats, or other mammals and is a suitable and safe research object in the laboratory (14). A 10K genomic island of *R. anatipestifer* was previously reported (15). RAP44, a strong bacteriophage of *R. anatipestifer* belonging to the Siphoviridae family of tailed phages, was isolated from the feces of healthy Chinese Muscovy ducks. The complete genome consists of 49,329 nucleotides of linear double-stranded DNA molecules with 80 open-reading frames. This study revealed the relationship between the newly discovered *R. anatipestifer* 50K genomic island and the *R. anatipestifer* phage RAP44. In our research, we focused on the relationship between bacterial genomic islands and phage genomes, and through comparative analysis, we found an evident evolutionary relationship between them. This is significant because phage integration promotes bacterial evolution and genomic diversity. We also investigated integrase that plays an essential role in integrating the phage genome into bacterial chromosomes. By studying the integration between host bacteria and phages, we provided new insights into the evolution of the genomes of *R. anatipestifer*.

## Materials and methods

### Bacterial strains, plasmids, and growth conditions

*Riemerella anatipestifer* ATCC11845 was purchased from the Microbial Preservation Center (Guangzhou, Guangdong), whereas *R. anatipestifer* RA-YM was obtained from a laboratory collection (16). The bacterial strains and plasmids used in this study are listed in Table 1. *R. anatipestifer* and *Escherichia coli* X7213 culture conditions have been previously described (17). When needed, spectinomycin and chloramphenicol antibiotics were used at a final concentration of 100 μg/ml.

**Table 1:** Bacterial strains and plasmids used in this study.

	**Description**	**Source or reference**
**Strains**		
*R. anatipestifer* YM	*R. anatipestifer* wild-type strain (serotype 1)	Lab collection
*R. anatipestifer* ATCC 11845	Standard strain of *R. anatipestifer*	Purchased at Guangzhou Microbial Preservation Center
phage RAP44	*R. anatipestifer* bacteriophage RAP44	NC_019490.1
*E.coli* X7213	Thi-1 thr-1 leuB6 glnV44 fhuA21 lacY1 recA1 RP4-2-Tc::Mu λpir ΔasdA4 Δzhf-2::Tn10	Lab collection
**Plasmids**		
pRE112	Suicide vector, sacB mobRP4 R6K ori Cm^R^.	Life Technology
pRE112-Spec-int	pRE112- RAint vector with Spec^R^.	This study

### Genomic island prediction in *R. anatipestifer* ATCC 11845

Our previous studies identified a 10K genomic island integrase function using IslandViewer 4. This study used IslandViewer 4 and the Phage Search Tool to predict genomic islands in the *R. anatipestifer* ATCC 11845 genome (PHAST, http://phast.wishartlab.com/) (18). Six genomic islands were predicted in *R. anatipestifer* ATCC 11845. We obtained the whole-genome data of *R. anatipestifer* from NCBI and used the Artemis Comparison Tool (ACT) to compare differences between prophage sequences and *R. anatipestifer* genomes (19).

### The 50K genomic island in *R. anatipestifer*

We conducted a comparative genomic analysis of the *R. anatipestifer* 50K genomic islands and the RAP44 genome. The ACT was used to compare the differences in the 50K genomic island of *R. anatipestifer*. Nucleotide sequence alignment was used to construct a phylogenetic tree using the neighbor-joining (NJ) method and was further analyzed using MEGA v6.06 software (17).

The primers used in this study are listed in Table 2. The existence of the 50K genomic island in *R. anatipestifer* ATCC11845, *R. anatipestifer* RA-YM, and 20 clinical isolates was determined by polymerase chain reaction (PCR) amplification of the integrase marker gene using the P1/P2 primer pair. Amplification was performed using 2× MonAmp^*TM*^ Taq Mix (Code No. RN03001M). We performed pre-denaturation at 95°C for 5 min, denaturation at 95°C for 15 s, annealing at 60°C for 15 s, and extension at 72°C for 90 s (extension time selected according to fragment size, 1,000 bp/min). The detailed method has been described in our previous article (15). The PCR products were separated and detected by 0.8% agarose gel electrophoresis. The integrase homologous amino acid sequences were identified by searching the GenBank database using BLASTX protein homology/analogy recognition (20).

**Table 2:** Primers used in this study.

**Name**	**Sequence (5^′^-3^′^)**
P1	TTGCTTTATAAAACAGGTCTTAAAGCACAAAAGTGCAC
P2	CTAAAAAGAAGGCAACTTTATTTTTTTAGCCTCTTCAAATC
P3	ATTCTGTGATATTTCAACTCCTTGACCGCTTC
P4	AGGCTCCAAGAGTTCTACGATGAAATTGGAAT
P5	TGGGCTGTTAAGCAACTCTCTTATGAAACAAG
P6	AGCCATTTGTCAAAATTACCACCACTGATGAC
P7	TCACGCGT AGGCTCCAAGAGTTCTACGATGAAATTGGAAT
P8	GTGCATGC CCAATTTTAAATACATCTGAAACCCAAATAAGCCG
Spec F	TGCGGTACC GTCATCAAAATTTTCATTCGTGGACAATAACG
Spec R	GTGCATGC GTCACCTTGCTTTTGAGTGCAATTCCCTAAAAC

The underlined sequences indicate the restriction sites.

### 50K genomic island integration and excision

Genomic island integration and excision were also determined using PCR amplification. P3/P4 and P5/P6 primer pairs were used for detecting the excision of the 50K genomic island. P3/P5 and P4/P6 primer pairs were used for detecting the integration of the 50K genomic island. The PCR products were separated and detected by 0.8% agarose gel electrophoresis. An integration and excision model of the 50K genomic island was predicted.

### Suicide recombinant vector integration into *R. anatipestifer*

Integration of the suicide recombinant vector into *R. anatipestifer* was performed according to our previously described method (17). *R. anatipestifer* no. 1 was selected as the receptor strain because it did not have the 50K genomic island but had a 19-bp attachment site attB (Supplementary Table S1). Integrase and the attP sites were amplified using the P7/P8 primer pair. A pRE112-Spec-int integration vector was constructed, and the detailed method is described in a published article (15). The pRE112-Spec-int plasmid was transformed into donor strain *E. coli* X7213. The *R. anatipestifer* integration strain was confirmed by PCR, and the integration and excision functions of the recombinant strains were verified. The genetic stability of the recombinant strain was detected. The recombinant bacteria regrew in the TSA pallet with Spec antibiotic after 20 passages without antibiotics.

## Results

### Comparative analysis of the 50K genomic island and RAP44 phage

**Figure 1:**
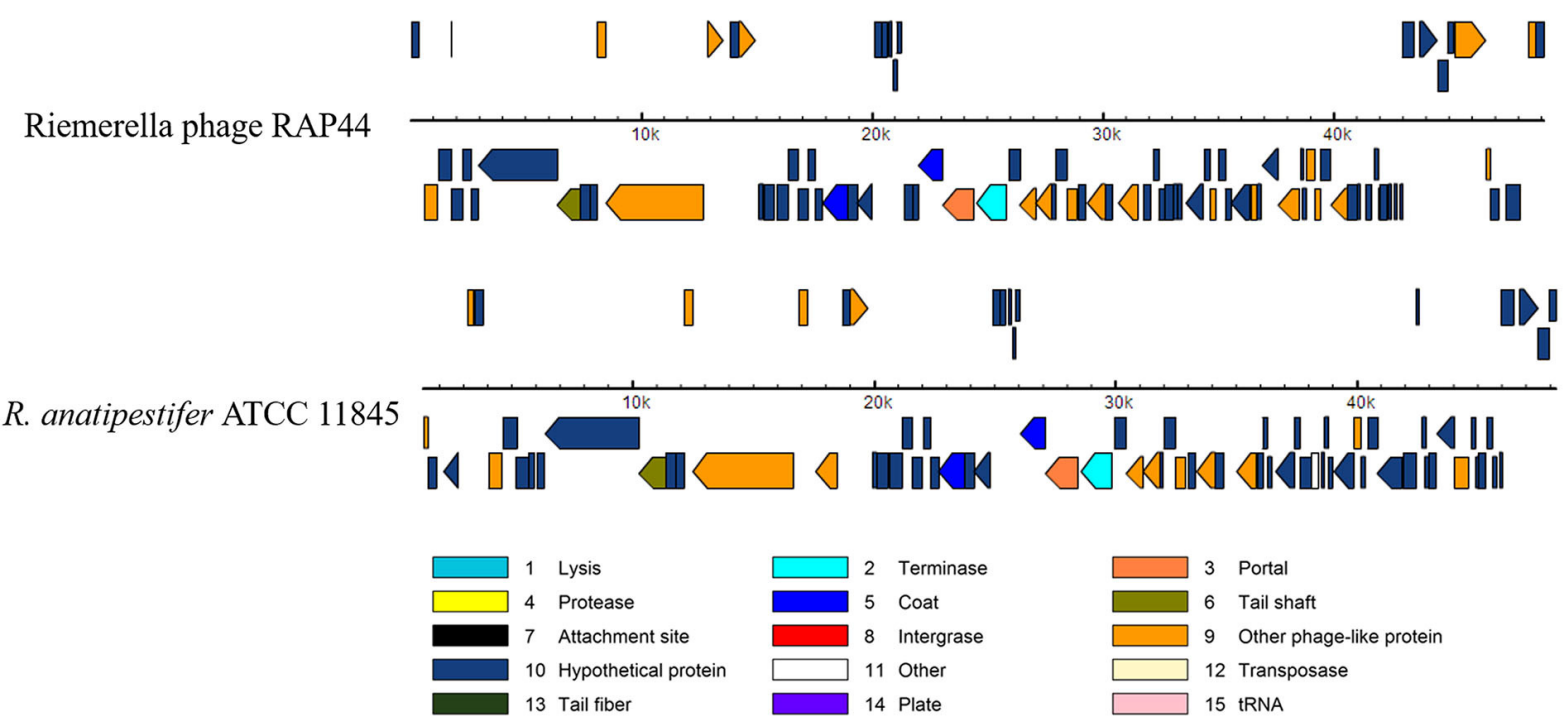
The 50K genomic island proteins related to RAP44 phage. PHAST was used to analyze 50K genomic island, and different phage proteins are shown.

**Figure 2:**
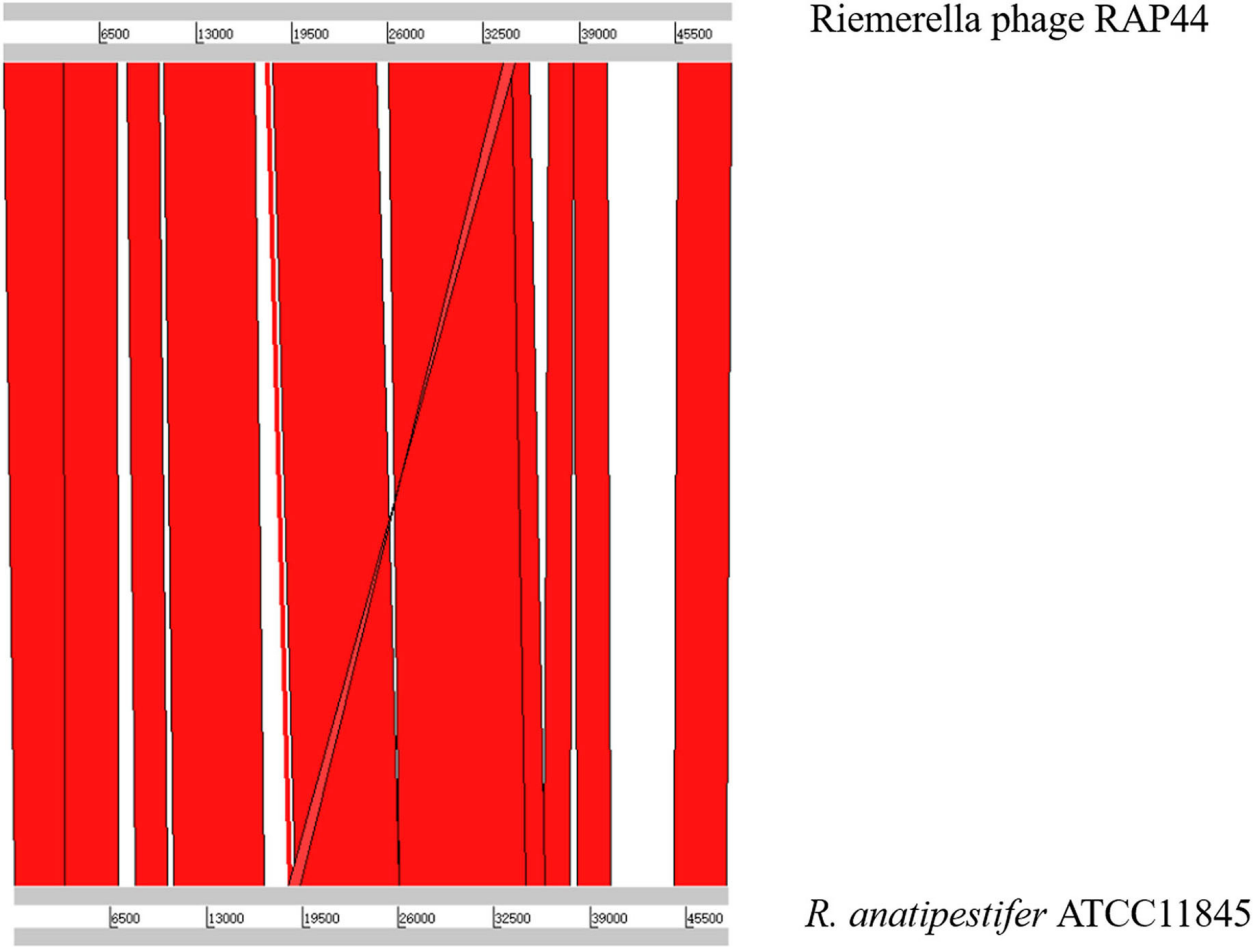
The relationship between 50K genomic island and RAP44 phage.

We selected the *R. anatipestifer* ATCC 11845 standard strain as our research target. The whole genome of *R. anatipestifer* ATCC 11845 has been published. IslandViewer 4 was used to predict bacterial GIs. There were six predicted genomic islands in *R. anatipestifer* ATCC 11845 (Supplementary Table S2). Among them, the 10K genomic island was reported in our previous articles. PHAST was used to predict prophages in *R. anatipestifer* ATCC 11845. There was a 50K intact prophage predicted by PHAST. The 50K prophage was also predicted to be a genomic island; thus, it was called the 50K genomic island and was highly homologous to the *R. anatipestifer* RAP44 phage. The 50K genomic island-coded protein predicted by PHAST is shown in Figure 1. Most proteins were RAP44 phage-related proteins. The results showed that the phage genomes were integrated into the bacterial genome. We also compared the differences between the 50K genomic island and the RAP44 phage. Large deletions and insertions were also identified when the phage genome and genomic island were analyzed using ACT (Figure 2).

### Analysis of the 50K genomic island structure in *R. anatipestifer*

**Figure 3:**
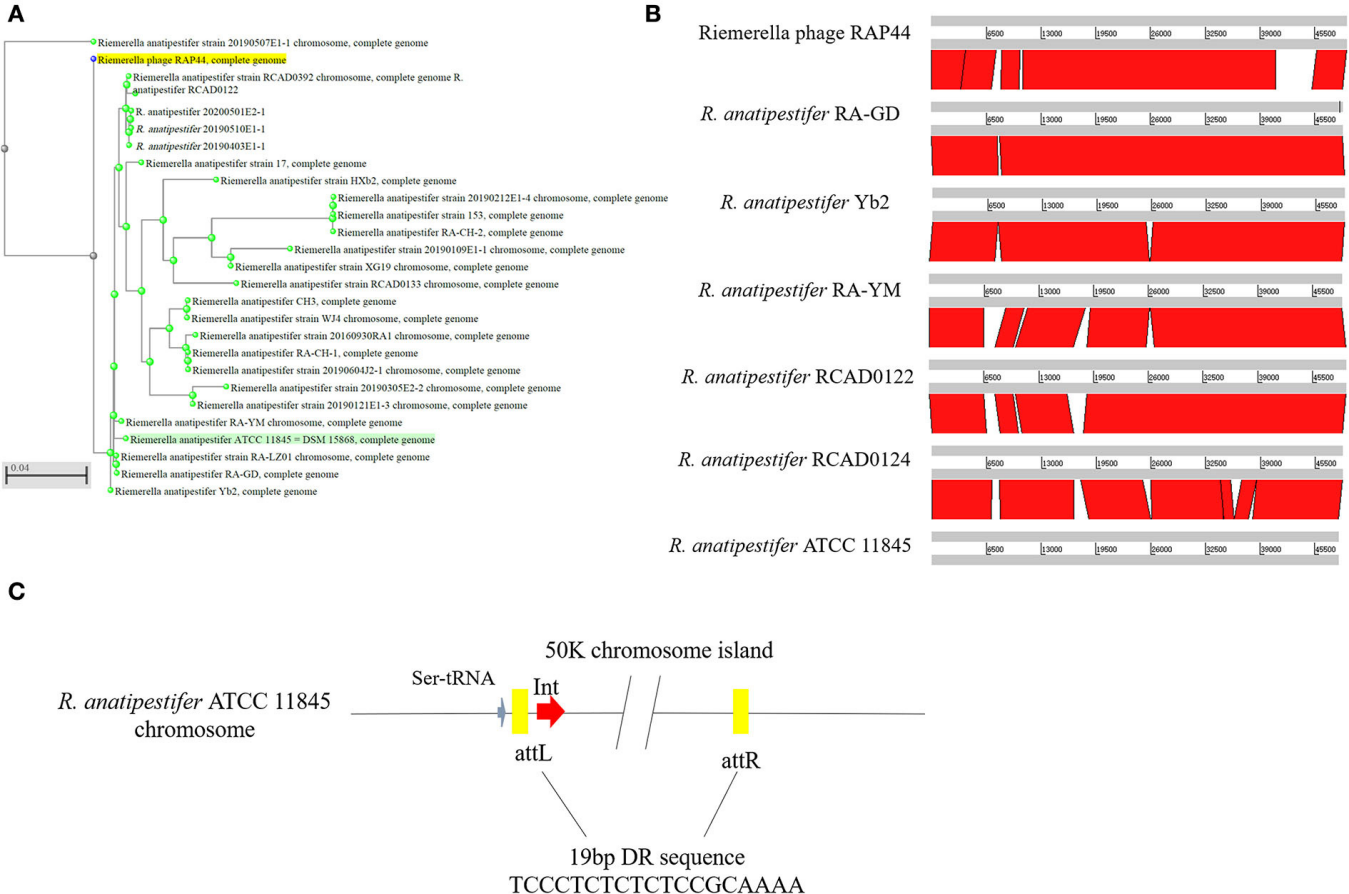
Evolution and components of 50K genomic island in *R. anatipestifer*. **(A)** Comparative genomic analysis of *R. anatipestifer* genome 50K genomic island and RAP44 genome. The nucleotide sequences alignment was used for the construction of a phylogenetic tree using the neighbor-joining (NJ). **(B)** The ACT for comparing the difference in 50K genomic islands from six *R. anatipestifer* strains. **(C)** The 50K genomic island simulation diagram. 50K genomic island located downstream of tRNA, and 19-bp repeat sequences (TCCCTCTCTCTCCGCAAAA) in the two flanks.

Whole-genome comparisons revealed that the RAP44 phage genome was integrated into the chromosome and formed a 50K genomic island in *R. anatipestifer*. Supplementary Table S3 shows the results of the homology comparison of the RAP44 and 50K genomic island genes. The replication proteins and other proteins of RAP44 were deleted, and several exogenous genes were inserted into *R. anatipestifer*. We performed a comparative genomic analysis between *R. anatipestifer* genomes and RAP44 phage genomes. Nucleotide sequence alignment was used to construct a phylogenetic tree using the NJ algorithm (Figure 3A). ACT was used to compare the differences between the 50K genomic island and RAP44 (Figure 3B). The prophages and genomic islands sequences had some common characteristics: (1) direct repeat sequences attL and attR were located on the two flanks of the genomic island and (2) integrase was located on the medial side of direct repeat sequences. The 50K genomic island was located downstream of transfer-ribonucleic acid (tRNA), and 19-bp repeat sequences (TCCCTCTCTCTCCGCAAAA) were present in the two flanks. A 50K genomic island simulation diagram was constructed (Figure 3C).

### Conservation analysis of 50K genomic island integrase

**Figure 4:**
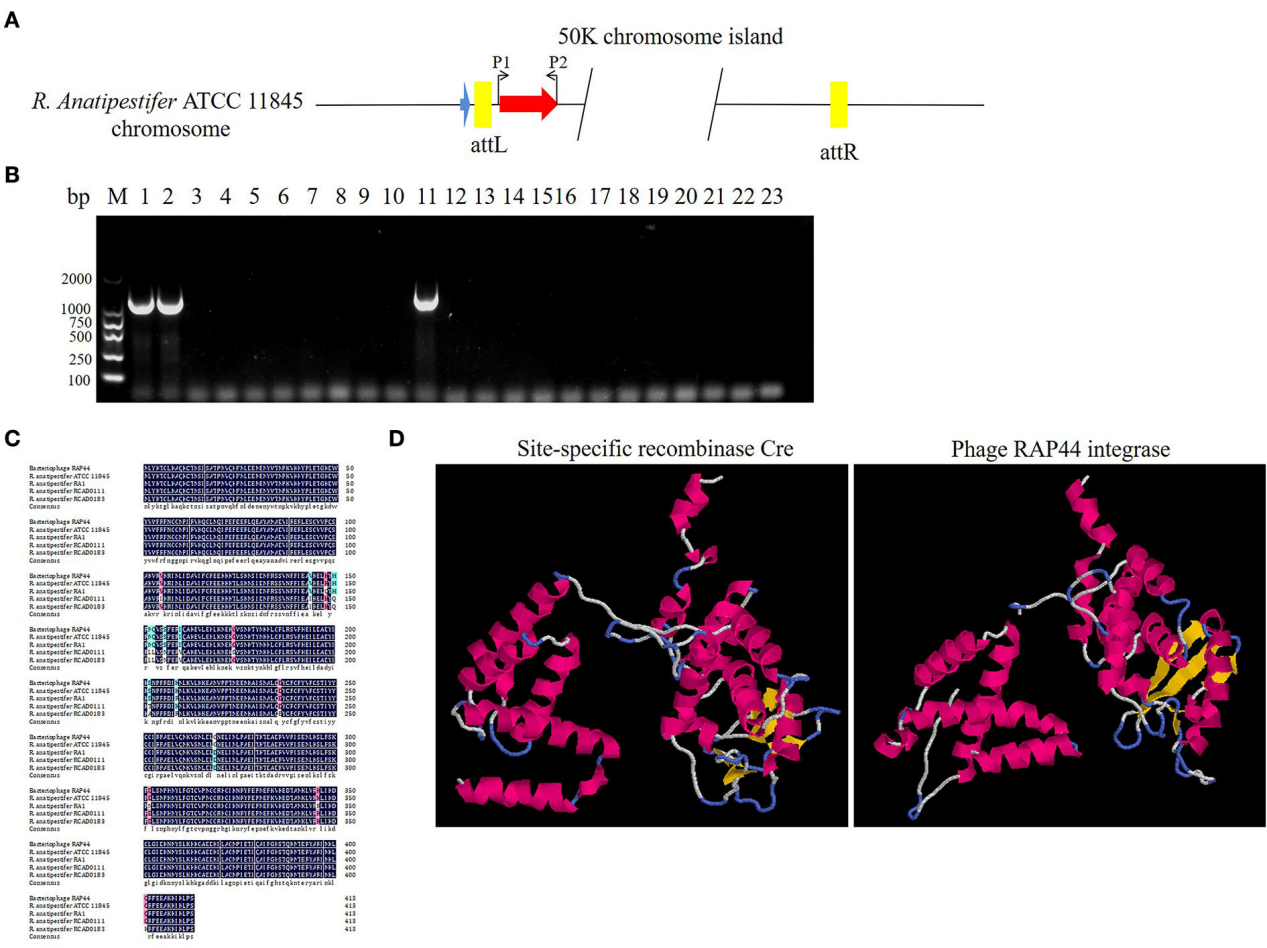
Analysis of RAP44 integrase and genomic island in *R. anatipestifer*. **(A)** Integrase gene with P1/P2 primer pairs was detected in *R. anatipestifer* ATCC 11845, RA-YM, and 20 *R. anatipestifer* clinical isolated strains. **(B)** Detecting of integrase in *R. anatipestifer* clinical isolated strain with primer pair P1/P2. Lane 1: *R. anatipestifer* ATCC11845 strain; lane 2, *R. anatipestifer* YM strain; lanes 3–22: 20 clinical isolated strains no. 1–20; lane 23: negative control; ATCC 11845, RA-YM, and clinical isolated strain no. 9 with the integrase. **(C)** Sequence alignment analysis shows that the integrase was tyrosine integrase and highly conserved in *R. anatipestifer*. **(D)** Phyre^2^ was applied to predict the integrase protein 3D structure. We found that the integrase protein structure is similar to tyrosine recombinase Cre.

The prophage was preferentially integrated into the tRNA gene through its encoded integrase, so its attachment site was tRNA, which was highly conserved and contributed to the genetic variation of bacteria. Integrase had high specificity and was highly efficient. Research has shown that prophage integrase typing is a useful indicator of genetic diversity in Salmonella enterica (21). RAP44 encodes a lambda repressor-like DNA-binding domain protein sequence, which is the same as that of *R. anatipestifer* tyrosine-type recombinase/integrase. In this study, the integrase gene was detected with the P1/P2 primer pairs in *R. anatipestifer* ATCC 11845, RA-YM, and 20 clinically isolated *R. anatipestifer* strains (Figure 4A). The results indicated ATCC 11845, RA-YM, and clinically isolated strain no. 9 with integrase gene (Figure 4B). Sequence alignment analysis showed that the integrase was a tyrosine integrase and was highly conserved in *R. anatipestifer* (Figure 4C). Three-dimensional structures of integrase protein (protein ID: YP_007003674.1) were predicted and modeled from the Phyre^2^ database. We found that it was similar to tyrosine recombinase Cre (protein ID: AAQ13978.1) (Figure 4D).

### 50K genomic island integration and excision

**Figure 5:**
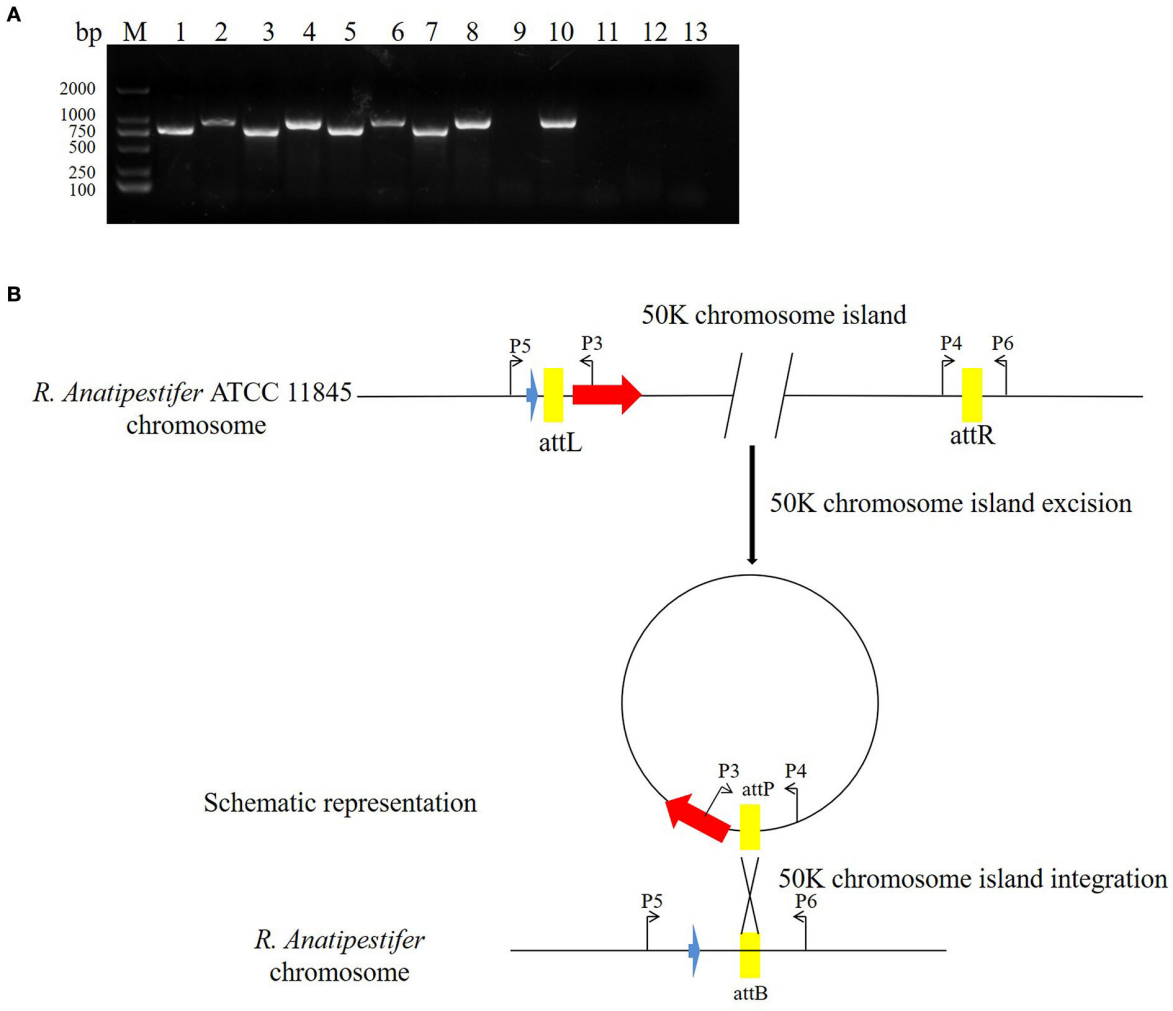
The 50K genomic island integration and excision. **(A)** The primer pairs P3/P4, P5/P6 were used for detecting genomic island excision, and P3/P5, P4/P6 were used for detecting genomic island integration in *R. anatipestifer* ATCC 11845, RA-YM, and clinical isolated strain no. 1. M: DL2000 DNA marker; lane 1: DNA fragment was amplified with P3/P4 primer pair from *R. anatipestifer* ATCC11845; lane 2: DNA fragment was amplified with P5/P6 primer pair from *R. anatipestifer* ATCC11845; lane 3: DNA fragment was amplified with P4/P6 primer pair from *R. anatipestifer* ATCC11845; lane 4: DNA fragment was amplified with P3/P5 primer pair from *R. anatipestifer* ATCC11845; lanes 5–8: DNA fragment was amplified with same primer from *R. anatipestifer* YM; lanes 9–12: DNA fragment was amplified with same primer from *R. anatipestifer* clinical isolated strain no. 1; lane 13: negative control; *R. anatipestifer* ATCC 11845 and RA-YM genomic islands had the integration and excision function. **(B)** Schematic representation of the integration and excision model.

The pathogenic island can be excised from the chromosome to form an unstable plasmid, replicating in *S. aureus* using its replicon (11). Three pathogenic islands of *Vibrio cholerae* (SPI-2, VSP-I, and VSP-II) can also be excised from the chromosome, forming circular intermediates (22). In this study, the primer pairs P3/P4 and P5/P6 were used to detect 50K genomic island excision. The primer pairs P3/P5 and P4/P6 were used to detect genomic island integration in *R. anatipestifer* ATCC 11845, RA-YM, and clinically isolated strain no. 1. *R. anatipestifer* ATCC 11845 and the RA-YM genomic islands had integration and excision functions (Figure 5A). This genomic island can be spontaneously excised and circularized from the *R. anatipestifer* chromosome. Figure 5B shows a schematic of the integration and excision models. Our research indicates that the 50K genomic island has a mobile function. The *R. anatipestifer* with this genomic island had chromosomal heterogeneity.

### 50K genomic island integrase-mediated integration in *R. anatipestifer*

**Figure 6:**
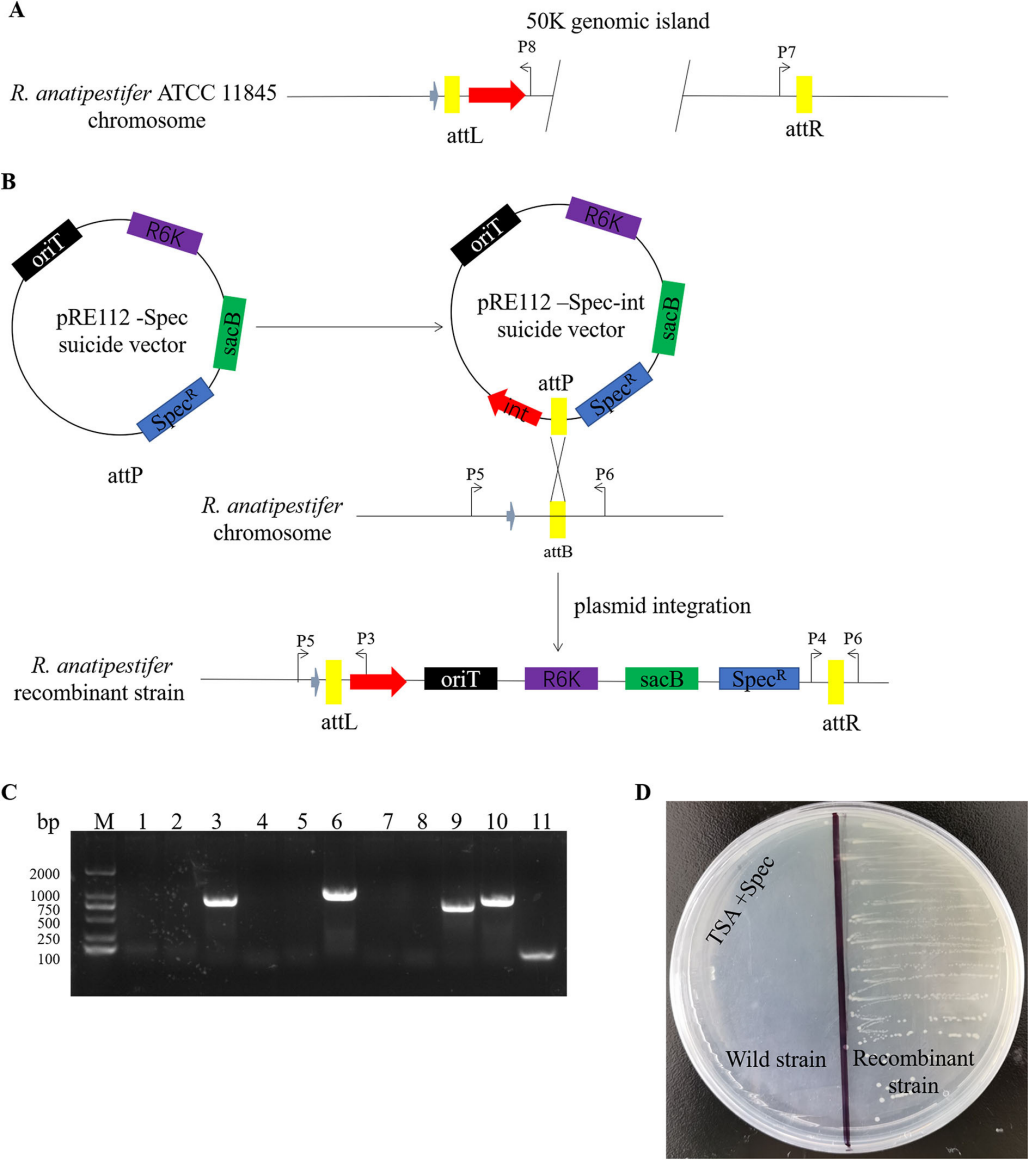
Identification of an integrase from phage RAP44-mediated integration in *R. anatipestifer*. **(A)** The integase with promoter and attP site was cloned with P7/P8 primer pairs. **(B)** The DNA fragments were cloned into suicide vector pRE112-Spec, the recombinant vector named pRE112-Spec-int. The construction diagram of integration vector is shown. **(C)** The recombinant strain was selected using Spec antibiotic plate and called *R. anatipestifer* RAint. Integration and excision were detected by PCR in the recombinant strain. M: DL2000 DNA marker; lane 1: *Spec* gene fragment was amplified with Spec F/R primer pair from *R. anatipestifer* clinical isolated strain no. 1; lane 2: DNA fragment was amplified with P3/P4 primer pair from *R. anatipestifer* clinical isolated strain no. 1; lane 3: DNA fragment was amplified with P5/P6 primer pair from *R. anatipestifer* clinical isolated strain no. 1; lane 4: DNA fragment was amplified with P4/P6 primer pair from *R. anatipestifer* clinical isolated strain no. 1; lane 5: DNA fragment was amplified with P3/P5 primer pair from *R. anatipestifer* clinical isolated strain no. 1; lanes 6–10: DNA fragment was amplified with same primer from recombinant strain; lane 11: negative control. Plasmid was integrated into *R. anatipestifer*, and the insert site was consistent with the *R. anatipestifer* ATCC 11845 and RA-YM. The recombinant strain only had the function of integration and loss of the function of excision. **(D)** Integrase-mediated integration has genetic stability. The well expression of Spec resistance cassette in recombinant bacteria after 20 passages in TSA pallet without antibiotic.

Here, we studied the integration mechanism of the genomic islands in *R. anatipestifer*. Integrase with the promoter and attP site was cloned with P7/P8 primer pairs and then connected with suicide vector pRE112-Spec (Figure 6A). The recombinant vector was named pRE112-Spec-int. The construction diagram of the integration vector is shown in Figure 6B. The recombinant suicide vector was transferred from the *E. coli* X7213 strain to the clinically isolated strain no. 1 *via* conjugal transfer. The recombinant strain was selected by Spec antibiotic plate and was called RAint. Integration and excision were detected by PCR of the recombinant strain. The results indicated that the plasmid was integrated into *R. anatipestifer*, and the insert site was consistent with *R. anatipestifer* ATCC 11845 and RA-YM. The recombinant strain only had the function of integration and lost its excision function (Figure 6C). Integrase-mediated integration is genetically stable. The recombinant bacteria after 20 passages in tryptone soybean agar pallet without antibiotics also have stable expression Spec-resistance cassette (Figure 6D). The recombinant strain was analyzed using high-throughput sequencing, and the insertion site and sequence were confirmed (Supplementary material). Our results indicate that the integrase from 50K genomic island mediated integration and stable protein expression in *R. anatipestifer*.

## Discussion

With super bacteria's increasing virulence and drug resistance, it is particularly important to understand the causes that drive bacterial evolution. Phages are vital gene-transfer particles. Phage transduction is considered the main cause of HGT between bacteria. HGT is the primary method for bacteria to acquire virulence and drug-resistance genes, which is of great significance in medicine (23). There are three modes of HGT, namely, transformation, conjugation, and transduction. Phage genomes are integrated into bacterial chromosomes with replication of the host chromosome-integrated phage genome passive replication. Phage transduction is the main driving force of microbial evolution (24).

In this study, the *R. anatipestifer* genomic islands and prophages were predicted using IslandViewer 4 and PHAST, and a 50K genomic island was identified (Figure 1). The 50K genomic island sequence alignment indicated that most sequences were consistent with the *R. anatipestifer* phage RAP44 (Figure 2). Phage RAP44 was the first virulent phage to be reported in 2012. RAP44 phage with double-stranded DNA and 80 coding sequences were identified (25). Further comparative genomic analysis revealed significant differences between 50K genomic islands of different *R. anatipestifer* strains and RAP44 phage genome (Figures 3A,B). The inserted phage genes resulted in host evolution, whereas the deleted phage genes promoted the loss of phage function. The deletion of the RAP44 phage-related genes and the insertion of foreign genes made it impossible to form complete phage particles. Insertion of the phage genome promoted the evolution of *R. anatipestifer*.

We analyzed the locus of the 50K genomic island and found that this genomic island is located downstream of tRNA, and the integrase is located downstream of tRNA. The 19 bp repeats sequence is located at the flanks of the genomic island. According to the above analysis, we confirmed the basic molecular characteristics of the genomic island (Figure 3C). We used integrase as the marker gene to identify the genomic island of clinically isolated strains and found that only one clinically isolated strain contains the 50K genomic island (Figure 4B). Phages are the most abundant organisms on earth and play a critical role in microbial evolution. They are the agents of HGT that form a vast network of biology that connects all bacterial genomes. Bacteria often require an exchange of genetic material to adapt quickly to the environment (26). Bacteriophages shape bacterial evolution in various ways; they increase bacterial diversity through the selective capture of species (27), promote HGT (28), and serve as a repository of genetic innovations (29). Phages are thought to be the primary source of genetic material for bacteria to produce new genes (30). We also found that other bacteria have similar genomic islands. *Enterobacter* phase mEp460 and *Pseudomonas* phase D3 were predicted as genomic islands in Gram-negative bacteria *E. coli* and *P. aeruginosa*, respectively. *Staphylococcus* phage IME1364_01, *Listeria* phase B025, and *Lactobacillus* phase phiJB were predicted as genomic islands in Gram-positive bacteria *S. aureus, L. monocytogenes*, and *Lactobacillus delbrueckii*, respectively. Therefore, the integration of phage genome-mediated HGT is ubiquitous in bacteria.

Prophages are parasitic bacterial viruses that can integrate into the bacterial genome by integrase, replicating on the host chromosome. Bacteria that integrate phage genes can produce additional virulence factors that enhance bacterial virulence and the ability to adapt and survive in various environments (31). HGT enhances the frequency of exchange between strains (32). Both exist in integrative and excisional forms of prophages in bacteria. Most of these exist in an integrative form (33). In addition to phages, pathogenic islands function in integrating and excising chromosomes. There are differences in the integrase amino acid sequence of the 50K genomic island and RAP44 in several sites (Figure 4C). We found that the 3D structure of the 50K genomic island integrase may be similar to lambda integrase Cre (Figure 4D). In the λ bacteriophage, λ integrase catalyzes the integration between attP and attB sites, forming attL and attR sites. SOS or mitomycin C can induce the excision–replication–packaging cycle of SaPIs. Almost all SaPIs have attL and attR direct repeats on both sides, which encode integrases and insert them in the same direction at specific chromosomal locations. Here, we described a 50K genomic island, a phage cluster, in *R. anatipestifer* ATCC 11845 and RA-YM. This genomic island can be spontaneously excised and circularized from the *R. anatipestifer* chromosome (Figure 5). Here, the SOS reaction was used to induce prophage of *R. anatipestifer* ATCC 11845 and RA-YM, and no phage particles were detected in the supernatant (data not shown). Prophage Spn1 of *Streptococcus pneumoniae* and PP3 of *P. aeruginosa* had similar characteristics.

Phage-derived integrases play an important role in gene expression in microorganisms, cells, plants, and animals. Stabilization of chromosomal integration of large DNA fragments is mediated by phage integrase in *Clostridium ljungdahlii* (34). A recent study showed that the BxB1-integrase system guided stable exogenous gene expression in *Pseudomonas* (35). In addition, λ integrase mediates a seamless vector transgenesis platform for therapeutic protein expression (36). Furthermore, a novel approach to plastid transformation uses phiC31 phage integrase (37). Cre/lox-mediated genome modifications have been widely used in mouse functional genomic research (38). We constructed the recombinant suicide plasmid containing integrase and attP site. The recombinant suicide plasmid had been integrated into clinical isolates of *R. anatipestifer* through conjugation transfer. The results indicated that integrase only possesses integrase activity, not possess exonuclease activity (Figure 6C). The recombinant strain obtained by resistance screening, and the foreign resistance gene were stable expression (Figure 6D). Integrase can catalyze the removal and reconnection of attL and attR loci. In addition to integrase, integration and excision reactions require the integration of host factors, and excision requires λ excisionase. The excision function of this genomic island may be mediated by exonuclease, and we will further explore the molecular mechanism of the excision function of this genomic island.

Phage-derived integrases can be used in various hosts. *Clostridium* phages reported in infections include *C. difficile* and pathogenic *C. perfringens*. The phage attachment site attP and bacterial attachment site attB have also been identified (39, 40). Moreover, the integrase systems of *Clostridium* phages have shown universality in different bacteria (41). They are functional and can be used as genetic manipulation tools for *Clostridium*. Therefore, further research is required as these integrase systems are expected to be widely used as genetic tools. We identified phage-derived integrases and analyzed their integrative characteristics of *R. anatipestifer*. Based on this, we propose a straightforward method for establishing stable expression of exogenous genes in *R. anatipestifer*.

In this study, by comparing the genomic islands with the whole-genome sequences of the RAP44 phage, we found a 50K genomic island originating from the phages. The inserted phage lost its characteristics through deletion and insertion, and the RAP44 phage genome was a part of the bacteria. Furthermore, the genomic islands contain numerous integrated mobile genetic elements. In *R. anatipestifer* ATCC 11845, the 50K genomic island was integrated, excised, and cyclized automatically. Integrases are essential elements of integration, and the integrative function of integrase was verified in *R. anatipestifer*; the integrase with the attP site could be stably integrated at the attB locus of the *R. anatipestifer* genome. Recombinant strains can stably inherit and express exogenous genes. We identified phage-derived integrase and analyzed their integrative characteristics. Based on this, we propose evidence for the evolution of *R. anatipestifer* genomes.

## Data availability statement

The original contributions presented in the study are included in the article/Supplementary material, further inquiries can be directed to the corresponding author/s.

## Author contributions

YY and YW: funding acquisition, supervision, validation, writing (review and editing), and resources. YW: investigation, visualization, and writing—original draft. JD, JR, LL, and JL: methodology. SN, YL, YZ, SG, FY, HM, and W-xT: project administration. All authors contributed to the article and approved the submitted version.

## Funding

This project was supported by the National Natural Science Foundation of China (No. 32202808), Shanxi Province Excellent Doctoral Work Award-Scientific Research Project Genomic Island Integration in Riemerella anatipestifer (Nos. SXBYKY2021007 and SXBYKY2021043), Start-up Fund for doctoral research of Shanxi Agricultural University (Nos. 2020BQ63 and 2021BQ08), Shanxi Province Colleges and Universities Science and Technology Innovation Project (Nos. 2021L159 and 2021L132), Shanxi 1331 Project (Grant No. 20211331-13), Key Research Development Program of Shandong Province (No. 2019JZZY010719), and Shanxi Agriculture Research System (2022-07).

## Conflict of interest

The authors declare that the research was conducted in the absence of any commercial or financial relationships that could be construed as a potential conflict of interest.

## Publisher's note

All claims expressed in this article are solely those of the authors and do not necessarily represent those of their affiliated organizations, or those of the publisher, the editors and the reviewers. Any product that may be evaluated in this article, or claim that may be made by its manufacturer, is not guaranteed or endorsed by the publisher.
