# Supplementary material for: RAP44 phage integrase-guided 50K genomic island integration in Riemerella anatipestifer
Source: Front Vet Sci. 2022 Nov 29;9:961354. doi: 10.3389/fvets.2022.961354 (PMC9745183; doi:10.3389/fvets.2022.961354)
Supplement: Supplementary file 1 [file Table_1.DOCX]

Supplemental Table Legends

Supplemental Table S2 Predicted genomic islands in *R. anatipestifer* ATCC 11845

| Start | End | Size | GI Prediction Method |
| --- | --- | --- | --- |
| 203357 | 213406 | 10049 | IslandPath-DIMOB |
| 1511956 | 1528421 | 16465 | IslandPath-DIMOB |
| 1798,076 | 1805579 | 7503 | IslandPath-DIMOB |
| 1905568 | 1934253 | 28685 | IslandPath-DIMOB |
| 96384 | 144684 | 48300 | Islander |
| 1513434 | 1528617 | 15183 | Islander |
