# Supplementary material for: RAP44 phage integrase-guided 50K genomic island integration in Riemerella anatipestifer
Source: Front Vet Sci. 2022 Nov 29;9:961354. doi: 10.3389/fvets.2022.961354 (PMC9745183; doi:10.3389/fvets.2022.961354)
Supplement: Supplementary file 2 [file Table_2.DOCX]

Supplemental Table Legends

Supplemental Table S3 Homology analysis of 50K genomic island and RAP44 phage protein

| RAP44 Gene Name | Hit 50K Genomic Island | Protein  Name |
| --- | --- | --- |
| F372_gp01 | 100% | hypothetical protein |
| F372_gp02 | 100% | N-acetylmuramoyl-L-alanine |
| F372_gp03 | 100% | hypothetical protein |
| F372_gp04 | 100% | hypothetical protein |
| F372_gp05 | 100% | hypothetical protein |
| F372_gp06 | 100% | hypothetical protein |
| F372_gp07 | 100% | DNA breaking-rejoining enzyme |
| F372_gp08 | 85% | hypothetical protein |
| F372_gp09 | 100% | hypothetical protein |
| F372_gp10 | 100% | hypothetical protein |
| F372_gp11 | 100% | hypothetical protein |
| F372_gp12 | 100% | hypothetical protein |
| F372_gp13 | 71% | putative phage tail protein |
| F372_gp14 | 100% | hypothetical protein |
| F372_gp15 | 100% | putative tape measure domain protein |
| F372_gp16 | 100% | hypothetical protein |
| F372_gp17 | 100% | hypothetical protein |
| F372_gp18 | 100% | hypothetical protein |
| F372_gp19 | 100% | hypothetical protein |
| F372_gp20 | 100% | hypothetical protein |
| F372_gp21 | 97% | hypothetical protein |
| F372_gp22 | 95% | hypothetical protein |
| F372_gp23 | 96% | putative head protein |
| F372_gp24 | 89% | hypothetical protein |
| F372_gp25 | 98% | hypothetical protein |
| F372_gp26 | NO | hypothetical protein |
| F372_gp27 | NO | hypothetical protein |
| F372_gp28 | 96% | phage head morphogenesis protein |
| F372_gp29 | 100% | phage portal protein |
| F372_gp30 | 100% | phage terminase large subunit |
| F372_gp31 | 100% | phage terminase small subunit |
| F372_gp32 | 100% | ParB-like nuclease |
| F372_gp33 | 100% | adenine nucleotide alpha hydrolase-like protein |
| F372_gp34 | 100% | hypothetical protein |
| F372_gp35 | 98% | hypothetical protein |
| F372_gp36 | 93% | hypothetical protein |
| F372_gp37 | 100% | bacteriophage lambda NinG protein |
| F372_gp38 | 95% | hypothetical protein |
| F372_gp39 | 98% | hypothetical protein |
| F372_gp40 | 100% | hypothetical protein |
| F372_gp41 | 100% | phage antirepressor protein |
| F372_gp42 | 100% | hypothetical protein |
| F372_gp43 | 100% | DNA-damage-inducible protein D |
| F372_gp44 | 100% | hypothetical protein |
| F372_gp45 | 100% | hypothetical protein |
| F372_gp46 | 100% | hypothetical protein |
| F372_gp47 | 100% | hypothetical protein |
| F372_gp48 | 100% | hypothetical protein |
| F372_gp49 | 100% | hypothetical protein |
| F372_gp50 | 100% | hypothetical protein |
| F372_gp51 | 100% | phage antirepressor protein |
| F372_gp52 | 100% | DNA-binding protein |
| F372_gp53 | 100% | hypothetical protein |
| F372_gp54 | 100% | hypothetical protein |
| F372_gp55 | 99% | hypothetical protein |
| F372_gp56 | 100% | hypothetical protein |
| F372_gp57 | 100% | Ig domain-containing protein |
| F372_gp58 | 99% | S-adenosyl-L-methionine-dependent methyltransferase |
| F372_gp59 | 100% | hypothetical protein |
| F372_gp60 | 97% | hypothetical protein |
| F372_gp61 | NO | hypothetical protein |
| F372_gp62 | NO | phage replication protein |
| F372_gp63 | NO | hypothetical protein |
| F372_gp64 | NO | single-strand binding protein |
| F372_gp65 | NO | HNH endonuclease |
| F372_gp66 | NO | hypothetical protein |
| F372_gp67 | NO | phage exonuclease |
| F372_gp68 | NO | hypothetical protein |
| F372_gp69 | 100% | DNA-binding protein |
| F372_gp70 | 100% | hypothetical protein |
| F372_gp71 | NO | hypothetical protein |
| F372_gp72 | 96% | hypothetical protein |
| F372_gp73 | 100% | hypothetical protein |
| F372_gp74 | 100% | hypothetical protein |
| F372_gp75 | 100% | hypothetical protein |
| F372_gp76 | 100% | hypothetical protein |
| F372_gp77 | 100% | DNA-binding protein |
| F372_gp78 | 100% | hypothetical protein |
| F372_gp79 | 100% | hypothetical protein |
| F372_gp80 | 100% | lambda repressor-like DNA-binding domain protein |
