# Supplementary material for: RAP44 phage integrase-guided 50K genomic island integration in Riemerella anatipestifer
Source: Front Vet Sci. 2022 Nov 29;9:961354. doi: 10.3389/fvets.2022.961354 (PMC9745183; doi:10.3389/fvets.2022.961354)
Supplement: Supplementary file 3 [file Table_3.DOCX]

GGAGGAATTTCAAATTTATCGGTGTCAGTATTGTTGAAAATATAATCAAATAATTGTAAATTAATTACATCATTTTCAAATGATTTTCCAGTTAATTTTTCTTCGTGATAATATATTCTTGCCATAAGTTTGTTTCAATATTTATCTTTTTGTAGCCATTTGTCAAAATTACCACCACTGATGACTAAGTCACAAATGAAGGGCAAGTTCAATTTAGCACTAATCAAGACAAATACTTTTTGGCTTCGCCGAACCACTTCGTTAGGTTTCAACGTCACTAAATTAAGCAAAATAGCAGAAATGAAAAGCATTTTATCATGAAATACGCCCTAATTTATATCATTACCTAAACTCCGTCTTTTGCAAATAATACATAGCGGTAGTTTCTTATTTATATAAACCCACTGTACATTACGGTATTAAGTAAAAAAAACATTCACTTTATTGATAAACAAAATGTTGTTTTAGCCACAAAACATTTGCAAATAAATAGCCTGAGTAAAATTTAAATATTGGACATTAGGAGTAGCGAAAATGGAATTTGTTTACTTTTGTTTATTTCATAAAAAAACCGATGAACAAAGTCTTTATTCATCGGCGTTTTGAGCAATTTTGCGGAGAGAGAGGGAAACTTTTTGACCATTTTTTGCCATTTTTGAAATTTTAATTTATTGATAATCAATATAGTTTTGTTTTGACTTTTTTTTAAATTTGTGCACTTTTGGTGTTTTGTCTAGGGGTATCTGTTACCCCAAAAATCCAATTAAAATCAATGCCATAAACTATTCCTATTTTGTGTATCTGCTCAACGGTAAAGTGTGCGTTAATGCCACTTATATCTTGTTTTTTAATTTTATTAAACCGTTGCTTGGAAATTCCAATTTCATCGTAGAACTCTTGGAGCCTACGCGTATGACAGTCTCCGGAAGACGGTTGTTGCGCACGTATTCGGTGAACGCACTATGGCGACGCTGGGGCGTCTTATGAGCCTGCTGTCACCCTTTGACGTGGTGATATGGATGACGGATGGCTGGCCGCTGTATGAATCCCGCCTGAAGGGAAAGCTGCACGTAATCAGCAAGCGATATACGCAGCGAATTGAGCGGCATAACCTGAATCTGAGGCAGCACCTGGCACGGCTGGGACGGAAGTCGCTGTCGTTCTCAAAATCGGTGGAGCTGCATGACAAAGTCATCGGGCATTATCTGAACATAAAACACTATCAATAAGTTGGAGTCATTACCAAAAGGTTAGGAATACGGTTAGCCATTTGCCTGCTTTTATATAGTTCATATGGGATTCACCTTTATGTTGATAAGAAATAAAAGAAAATGCCAATAGGATATCGGCATTTTCTTTTGCGTTTTTATTTGTTAACTGTTAATTGTCCTTGTTCAAGGATGCTGTCTTTGACAACAGATGTTTTCTTGCCTTTGATGTTCAGCAGGAAGCTGGGCGCAAACGTTGATTGTTTGTCTGCGTAGAATCCTCTGTTTGTCATATAGCTTGTAATCACGACATTGTTTCCTTTCGCTTGAGGTACAGCGAAGTGTGAGTAAGTAAAGGTTACATCGTTAGGATCAAGATCCATTTTTAACACAAGGCCAGTTTTGTTCAGCGGCTTGTATGGGCCAGTTAAAGAATTAGAAACATAACCAAGCATGTAAATATCGTTAGACGTAATGCCGTCAATCGTCATTTTTGATCCGCGGGAGTCAGTGAACAGATACCATTTGCCGTTCATTTTAAAGACGTTCGCGCGTTCAATTTCATCTGTTACTGTGTTAGATGCAATCAGCGGTTTCATCACTTTTTTCAGTGTGTAATCATCGTTTAGCTCAATCATACCGAGAGCGCCGTTTGCTAACTCAGCCGTGCGTTTTTTATCGCTTTGCAGAAGTTTTTGACTTTCTTGACGGAAGAATGATGTGCTTTTGCCATAGTATGCTTTGTTAAATAAAGATTCTTCGCCTTGGTAGCCATCTTCAGTTCCAGTGTTTGCTTCAAATACTAAGTATTTGTGGCCTTTATCTTCTACGTAGTGAGGATCTCTCAGCGTATGGTTGTCGCCTGAGCTGTAGTTGCCTTCATCGATGAACTGCTGTACATTTTGATACGTTTTTCCGTCACCGTCAAAGATTGATTTATAATCCTCTACACCGTTGATGTTCAAAGAGCTGTCTGATGCTGATACGTTAACTTGTGCAGTTGTCAGTGTTTGTTTGCCGTAATGTTTACCGGAGAAATCAGTGTAGAATAAACGGATTTTTCCGTCAGATGTAAATGTGGCTGAACCTGACCATTCTTGTGTTTGGTCTTTTAGGATAGAATCATTTGCATCGAATTTGTCGCTGTCTTTAAAGACGCGGCCAGCGTTTTTCCAGCTGTCAATAGAAGTTTCGCCGACTTTTTGATAGAACATGTAAATCGATGTGTCATCCGCATTTTTAGGATCTCCGGCTAATGCAAAGACGATGTGGTAGCCGTGATAGTTTGCGACAGTGCCGTCAGCGTTTTGTAATGGCCAGCTGTCCCAAACGTCCAGGCCTTTTGCAGAAGAGATATTTTTAATTGTGGACGAATCGAACTCAGGAACTTGATATTTTTCATTTTTTTGCTGTTCAGGGATTTGCAGCATATCATGGCGTGTAATATGGGAAATGCCGTATGTTTCCTTATATGGCTTTTGGTTCGTTTCTTTCGCAAACGCTTGAGTTGCGCCTCCTGCCAGCAGTGCGGTAGTAAAGGTTAATACTGTTGCTTGTTTTGCAAACTTTTTGATGTTCATCGTTCATGTCTCCTTTTTTATGTACTGTGTTAGCGGTCTGCTTCTTCCAGCCCTCCTGTTTGAAGATGGCAAGTTAGTTACGCACAATAAAAAAAGACCTAAAATATGTAAGGGGTGACGCCAAAGTATACACTTTGCCCTTTACACATTTTAGGTCTTGCCTGCTTTATCAGTAACAAACCCGCGCGATTTACTTTTCGACCTCATTCTATTAGACTCTCGTTTGGATTGCAACTGGTCTATTTTCCTCTTTTGTTTGATAGAAAATCATAAAAGGATTTGCAGACTACGGGCCTAAAGAACTAAAAAATCTATCTGTTTCTTTTCATTCTCTGTATTTTTTATAGTTTCTGTTGCATGGGCATAAAGTTGCCTTTTTAATCACAATTCAGAAAATATCATAATATCTCATTTCACTAAATAATAGTGAACGGCAGGTATATGTGATGGGTTAAAAAGGATCAGAATTCCCATGTCAGCCGTTAAGTGTTCCTGTGTCACTCAAAATTGCTTTGAGAGGCTCTAAGGGCTTCTCAGTGCGTTACATCCCTGGCTTGTTGTCCACAACCGTTAAACCTTAAAAGCTTTAAAAGCCTTATATATTCTTTTTTTTCTTATAAAACTTAAAACCTTAGAGGCTATTTAAGTTGCTGATTTATATTAATTTTATTGTTCAAACATGAGAGCTTAGTACGTGAAACATGAGAGCTTAGTACGTTAGCCATGAGAGCTTAGTACGTTAGCCATGAGGGTTTAGTTCGTTAAACATGAGAGCTTAGTACGTTAAACATGAGAGCTTAGTACGTGAAACATGAGAGCTTAGTACGTACTATCAACAGGTTGAACTGCTGATCTTCAGATCCTCTACGCCGGACGCATCGTGGCCGGATCGATCCAGCCGACCAGGCTTTCCACGCCCGCGTGCCGCTCCATGTCGTTCGCGCGGTTCTCGGAAACGCGCTGCCGCGTTTCGTGATTGTCACGCTCAAGCCCGTAGTCCCGTTCGAGCGTCGCGCAGAGGTCAGCGAGGGCGCGGTAGGCCCGATACGGCTCATGGATGGTGTTTCGGGTCGGGTGAATCTTGTTGATGGCGATATGGATGTGCAGGTTGTCGGTGTCGTGATGCACGGCACTGACGCGCTGATGCTCGGCGAAGCCAAGCCCAGCGCAGATGCGGTCCTCAATCGCGCGCAACGTCTCCGCGTCGGGCTTCTCTCCCGCGCGGAAGCTAACCAGCAGGTGATAGGTCTTGTCGGCCTCGGAACGGGTGTTGCCGTGCTGGGTCGCCATCACCTCGGCCATGACAGCGGGCAGGGTGTTTGCCTCGCAGTTCGTGACGCGCACGTGACCCAGGCGCTCGGTCTTGCCTTGCTCGTCGGTGATGTACTTCACCAGCTCCGCGAAGTCGCTCTTCTTGATGGAGCGCATGGGGACGTGCTTGGCAATCACGCGCACCCCCCGGCCGTTTTAGCGGCTAAAAAAGTCATGGCTCTGCCCTCGGGCGGACCACGCCCATCATGACCTTGCCAAGCTCGTCCTGCTTCTCTTCGATCTTCGCCAGCAGGGCGAGGATCGTGGCATCACCGAACCGCGCCGTGCGCGGGTCGTCGGTGAGCCAGAGTTTCAGCAGGCCGCCCAGGCGGCCCAGGTCGCCATTGATGCGGGCCAGCTCGCGGACGTGCTCATAGTCCACGACGCCCGTGATTTTGTAGCCCTGGCCGACGGCCAGCAGGTAGGCCGACAGGCTCATGCCGGCCGCCGCCGCCTTTTCCTCAATCGCTCTTCGTTCGTCTGGAAGGCAGTACACCTTGATAGGTGGGCTGCCCTTCCTGGTTGGCTTGGTTTCATCAGCCATCCGCTTGCCCTCATCTGTTACGCCGGCGGTAGCCGGCCAGCCTCGCAGAGCAGGATTCCCGTTGAGCACCGCCAGGTGCGAATAAGGGACAGTGAAGAAGGAACACCCGCTCGCGGGTGGGCCTACTTCACCTATCCTGCCCGGCTGACGCCGTTGGATACACCAAGGAAAGTCTACACGAACCCTTTGGCAAAATCCTGTATATCGTGCGAAAAAGGATGGATATACCGAAAAAATCGCTATAATGACCCCGAAGCAGGGTTATGCAGCGGAAAAGCGCTGCTTCCCTGCTGTTTTGTGGAATATCTACCGACTGGAAACAGGCAAATGCAGGAAATTACTGAACTGAGGGGACAGGCGAGAGACGATGCCAAAGAGCTACACCGACGAGCTGGCCGAGTGGGTTGAATCCCGCGCGGCCAAGAAGCGCCGGCGTGATGAGGCTGCGGTTGCGTTCCTGGCGGTGAGGGCGGATGTCGAGGCGGCGTTAGCGTCCGGCTATGCGCTCGTCACCATTTGGGAGCACATGCGGGAAACGGGGAAGGTCAAGTTCTCCTACGAGACGTTCCGCTCGCACGCCAGGCGGCACATCAAGGCCAAGCCCGCCGATGTGCCCGCACCGCAGGCCAAGGCTGCGGAACCCGCGCCGGCACCCAAGACGCCGGAGCCACGGCGGCCGAAGCAGGGGGGCAAGGCTGAAAAGCCGGCCCCCGCTGCGGCCCCGACCGGCTTCACCTTCAACCCAACACCGGACAAAAAGGATCGGGCCCTAATACCTGTGACGGAAGATCACTTCGCAGAATAAATAAATCCTGGTGTCCCTGTTGATACCGGGAAGCCCTGGGCCAACTTTTGGCGAAAATGAGACGTTGATCGGCACGTAAGAGGTTCCAACTTTCACCATAATGAAATAAGATCACTACCGGGCGTATTTTTTGAGTTATCGAGATTTTCAGGAGCTAAGGAAGCTAAAATGGAGAAAAAAATCACTGGATATACCACCGTTGATATATCCCAATGGCATCGTAAAGAACATTTTGAGGCATTTCAGTCAGTTGCTCAATGTACCTATAACCAGACCGTTCAGCTGGATATTACGGCCTTTTTAAAGACCGTAAAGAAAAATAAGCACAAGTTTTATCCGGCCTTTATTCACATTCTTGCCCGCCTGATGAATGCTCATCCGGAATTCCGTATGGCAATGAAAGACGGTGAGCTGGTGATATGGGATAGTGTTCACCCTTGTTACACCGTTTTCCATGAGCAAACTGAAACGTTTTCATCGCTCTGGAGTGAATACCACGACGATTTCCGGCAGTTTCTACACATACATTCGCAAGATGTGGCGTGTTACGGTGAAAACCTGGCCTATTTCCCTAAAGGGTTTATTGAGAATATGTTTTTCGTCTCAGCCAATCCCTGGGTGAGTTTCACCAGTTTTGATTTAAACGTGGCCAATATGGACAACTTCTTCGCCCCCGTTTTCACCATGGCAGTAGTTTTAAAAGTAAGCACCTGTTATTGCAATAAAATTAGCCTAATTGAGAGAAGTTTCTATAGAATTTTTCATATACTTAACGAGTGCTTTTACCTTTGAATATAGTCCTTCCCACTTATCATCACACTCTCCCCGATAGCCTTTTCTAGCTATATCCAGTAAAGTTACATGCTCTTTAGGTAAAAGAGGTATAGCCCATTCTGCAGCGACATCTTTCGAGGTAATTTCACCAGTAGTCACTGTTTGCCACATTCGAGCTAGGGTTAAAATTACATTACGCTCATCACCTTTTATCCCCTCAATTAGTTCTGGCAAGGAATCCTTAATTGCTCTTCGAATATCTGTCAAAGGTACGGAGACAAGTATACTTGAAGAATCAGGACCAAATAGAGAAATACTATTCTTTCTTGCTTGTGCTAAAACAATAGCCAAATCAGGATCATAGCTTGGTTCCTGAATTTGTCCATTCTCAAATTCACCCCTGAGCCACTCACCGTATATAAATTCTCTTTTTGGAGGATATTGCCAAGGGACAACTTCACTCCTATTTATAACCGTAACTTCAAGTGGTCTAACAGAATCCGTATTTCCAATCTTTCCTGATATAGTCATTAGTCTTTCTGTTAGTTTTTTTCGAGTTAATTGAGGTAAACCATGATTCACGACGACTAGAACATCTACATCGCTGTTAATGCGTAAACCACCATTTACTGCTGAACCAAATAGATATACTCCAACTATTGAACCTCCAAATAAATCTTTTACGATTTTTAATGTTCGAATCGCTTGATTTGGTATTTTTCCGTTAATCAAATTGCTCATGATTTCACCTCGTTGATTATGTTCATATAAAGTTTATATTGATACTCAATTTACTTACCCTAGATTGGACATATACTTAGATTACTGTTCAATAAAGCTGACCGTTAGCGTTTAAGTACATCCTTTCACAATTTGTCTACAGATTAATAATTATTCTTTATTATACAGATCGATCCTCTAGAATAAAAGGATCTAGGTGAAGATCCTTTTTGATAATCTCATGACCAAAATCCCTTAACGTGAGTTTTCGTTCCACTGGCATGCCCAATTTTAAATACATCTGAAACCCAAATAAGCCGTTATAAAAAAGGCAAGACCGAAATAACAGTTGCTTTATTAAAAAAATGGTGTGAAATCCTACAAATAGATATTAAAAAACTTTTTAATTGAAAAACCGCGAGAACTTTGTAAATTCCCGCGGTTTTTATCTAAAAAGAAGGCAACTTTATTTTTTTAGCCTCTTCAAATCTTTGTAGTTTCATTATCCTAGCGTATCTCTCTGTCATTTTTTGGGTAGAGTGTCCAAATATAGCCTGTATAGTTTCAATAGGCATTCCTGCTAGTATTTTATCATCCGCTCCTTTGTGCTTCAAGGAGTACATATTTTTGTCAATGCCTAAGCCGTCTTTTATTAGCTTACGCCATAGTTTATTAGCTGTATCTTCCTTTACTTTGAACTCATTGGGCTCAAAATATCTATTTTTAATACCGTGTCTTCCTCCATTCGGCACGCAGGTACCAAACAAATAGTTATGAGGGTTAGAAAGGTCAAATTTTGAAAATAGACTTTTCAAGTTTTCGCTTATAGGTACTACCCTATCAGCATCTGTTTTGGTTATTTCAGCAGGTAAATTGATAAGCTCGTTGCCCAAATCTAGCATTGACACTTTCATTTGCACCAATTCCGCAGGACGGATACCGCAATAGTAGATAGTGGAGCAAAATACATAGAATCCAAAACAGTAATCCTGCAAAGCGTTAGATATAGCTTTCATTTCCTCATTTGTCGGAGGTACATTTGCCTCTTTTTTTAAAACCTTTAAATTCTTAATATCCCTAAACGGATTACTTTTTATGTAGTCAGCATCAAGTATTTCGTGAAAAACAGACCTTAAAAAGCCAAGATGCTTATTGTAAGTCTTGTTAGACCAGCCTTTCTCATTTTTTAGATGTTCCAGAACCTCTTTAGCCTGTATCCTTTCAAAACTAGACACCTGCATTCTGTGATATTTCAACTCCTTGACCGCTTCAATAAAGAAATTGACAGAGCTTCTAAAATTATCAATACTATTTTTTGAAAGAGTTTTCTTTTTTTCCTCAAAGCCAAATATAACGGCATCTATAAGATTTATCCTTTTTACCCTCACTTTAGCACTTTGAGGAACCCAACCACTCTCTAGCCTTTCTCTTATGACATCTGCCATTGCATAAGCCTCTTGCAGCCTTTCCTCAAATTCTGGAATCTGATTAAGCCCTTGCTTTACTCTAATGGGATTACCTCCGTTAAATCGAAACCAAACATACCAATCCTTACCTGTTTCTAAGGGGTAATGTTTCACTTTGGGATTAGTCCAATAATTTTCCATTTCGTCCAAATTAAAGTGTTGGACATTAGGGGTAGCGGAAATGGAATTTGTGCACTTTTGTGCTTTAAGACCTGTTTTATAAAGCAAAACCGTTGATTTACAACGGCTTAACTCCTTTTTGCGGAGAGAGAGGGATTCGAACCCCCGGACCTGTTACAGTCAACGGTTTTCAAGACCGCCGCATTCGACCGCTCTGCCATCTCTCCTTAACAACTCTATCGTTGTTTTTAGTGGTGCAAATATATAACTATTTTTAATTATACACAAATTTTACATTCACTTTCAACTCCAAAATAGTATAAATTACTATTTATCAATAATAAAAATTTAGACTACCCTACTTTAAATCCTAAATAAACTGCCCAAAATCCTAATACTAAACTTGCCACCACATAGCTAAAAAGAGTTACATAATCCTGATTTTGCCATAACACAAGGTTCTCGTAGGAAAAAGTAGAAAATGTAGTAAAACCTCCGCACAGCCCCACTATAAATAAATATCTAATAATATCAGATTCTTGTTTCATAAGAGAGTTGCTTAACAGCCCAATAAAAAAACAACCCAATACATTAACCACCAATGTCCCCATAGGAAAAGTCCCTAATTTGAAAAACTGAGCGGTATAACGAGAAACAAGGAAGCGAAATACACTTCCTAATCCTCCGCCTATGAATATATATAGTATTGTTTTCATTTTGTATATAACGAGTGCTGAAACTAGCCCCGATGGAAGCGGATACCCCGCAGTGAAGCCGCGGAACGAAGTGGAGCGGCAAAACGAGGAGTATGAGCGGACAGCGGGAAATAGCTCCTAAAAAATAGAACCTATTTCCGCTCCGCTTTTTAATGATTATCCTTTTAATTCC
